# The Stiffness Variation of a Micro-Ring Driven by a Traveling Piecewise-Electrode

**DOI:** 10.3390/s140917256

**Published:** 2014-09-16

**Authors:** Yingjie Li, Tao Yu, Yuh-Chung Hu

**Affiliations:** 1 School of Electromechanical Automobile Engineering, Yantai University, Yantai 264005, China; E-Mails: liyingjie107@126.com (Y.L); yt_126@126.com (T.Y.); 2 Department of Mechanical and Electromechanical Engineering, National ILan University, 26041 ILan, Taiwan

**Keywords:** electrostatics, MEMS, microstructures, stabilities, stiffness

## Abstract

In the practice of electrostatically actuated micro devices; the electrostatic force is implemented by sequentially actuated piecewise-electrodes which result in a traveling distributed electrostatic force. However; such force was modeled as a traveling concentrated electrostatic force in literatures. This article; for the first time; presents an analytical study on the stiffness variation of microstructures driven by a traveling piecewise electrode. The analytical model is based on the theory of shallow shell and uniform electrical field. The traveling electrode not only applies electrostatic force on the circular-ring but also alters its dynamical characteristics via the negative electrostatic stiffness. It is known that; when a structure is subjected to a traveling constant force; its natural mode will be resonated as the traveling speed approaches certain critical speeds; and each natural mode refers to exactly one critical speed. However; for the case of a traveling electrostatic force; the number of critical speeds is more than that of the natural modes. This is due to the fact that the traveling electrostatic force makes the resonant frequencies of the forward and backward traveling waves of the circular-ring different. Furthermore; the resonance and stability can be independently controlled by the length of the traveling electrode; though the driving voltage and traveling speed of the electrostatic force alter the dynamics and stabilities of microstructures. This paper extends the fundamental insights into the electromechanical behavior of microstructures driven by electrostatic forces as well as the future development of MEMS/NEMS devices with electrostatic actuation and sensing.

## 1. Introduction

The principle of electrostatic force is very commonly used for the micro actuating/sensing devices in Micro/Nano ElectroMechanical Systems (MEMS/NEMS). The electrostatic driving principle consists of the coupling of two energy domains: electrical and mechanical energy domains. It is very challenging to accurately model the electrostatic microstructures because of the nonlinear electromechanical coupling behavior [1–3]. Chuang *et al.* [4] had published a review article which surveyed 132 literatures about the techniques for the physical model of pull-in voltage, dynamic characteristic analysis, air damping effect, reliability, numerical modeling method, and application of electrostatic-driven MEMS devices. Furthermore, the effects of non-ideal boundary conditions, fringing fields, the pre-deformation induced by initial stresses, and non-homogeneous structures were also detailed in that review paper. Another review article written by Zhang *et al.* [5] surveyed 341 literatures on various state-of-the-art approaches for the pull-in instability and further enhancing the performance of MEMS/NEMS devices with electrostatic actuation and sensing as well as the physical principles that have enabled fundamental insights into the pull-in instability. Micro-ring resonators play an important role in silicon photonics [6–8] and metamaterials [9,10]. After surveying the massive correlative literatures mentioned in the aforesaid literatures, there is no literature investigating the electromechanical behavior of a microstructure driven by traveling electrostatic forces. In the last couple of years, Hu *et al.* had extended his interest on the fundamental understanding of the electromechanical behavior of microstructures (micro-ring [11] and micro-beam [12]) driven by traveling electrostatic forces. In those two previous works, the traveling electrostatic force was modeled as a concentrated electrostatic force traveling on the microstructure. However, the model of concentrated traveling electrostatic force deviates somewhat from practice because the electrostatic force is implemented by distributed driving electrode in practice. Therefore, this paper aims to derive an analytical model for simulating a micro-ring driven by a traveling piecewise-electrode. The stiffness variation of the micro-ring affected by the traveling speed, driving voltage, and length of the piecewise-electrode are investigated in the present paper.

## 2. Analytical Model

**Figure 1.**
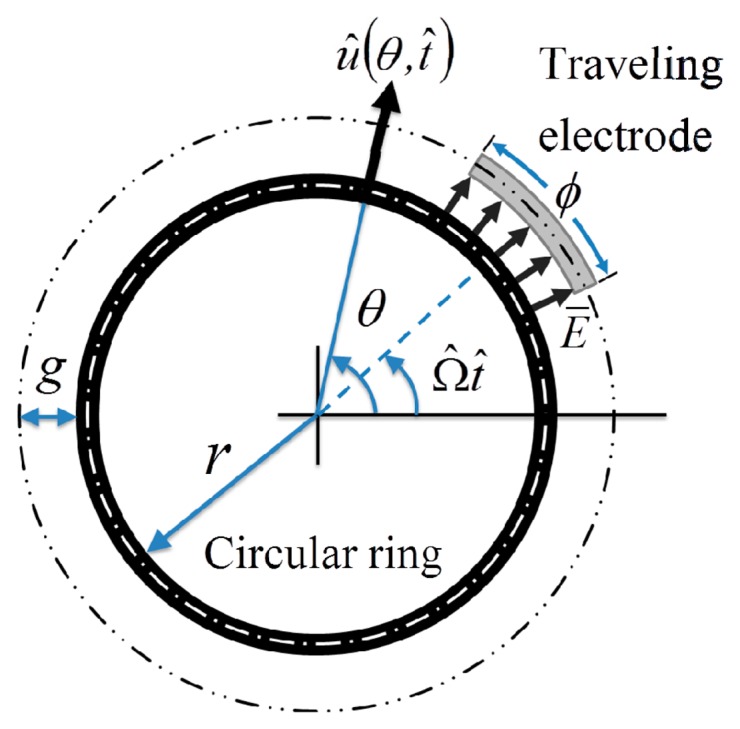
The schematic diagram of a micro circular-ring around which goes an arc-type electrode.

An analytical model is derived for simulating the electromechanical behavior of a micro circular-ring around which goes an arc electrode whose width is the same as that of the circular-ring and span angle *ϕ*. The schematic diagram is shown in Figure 1. The circular-ring is isotropic, homogeneous and of constant thickness. The analytical model is based on the assumptions that the circular-ring is thin with respect to its radius and that deflection is reasonably small, and furthermore considers only the uniform electrical field between the circular-ring and the traveling electrode. On these three basic assumptions, the circumferential inertia of the circular-ring can be assumed to be negligible because there is no circumferential force. If the planar dimension of the structure is much larger than the air gap between structure and substrate, the structure would be affected by the air damping during movement. However, this work focuses on the circular ring structure whose planar dimension is not much larger than the air gap and thus the air damping effect is negligible in this work.

### 2.1. Nomenclature


*A*The cross-sectional area of circular-ring*b*The widths of circular-ring and traveling arc electrode*E*The Young's modulus of circular-ring*E̅*The uniform electrical field in between circular-ring and traveling electrode*g*The initial gap between circular-ring and traveling arc electrode*H*(*θ*)The unit step function of *θ**I*The area inertia moment of the cross-section of circular-ring[**K**]The stiffness matrix[**K***^c^*]The electrostatic stiffness matrix due to traveling electrostatic force[**K***^s^*]The structural stiffness matrix due to circular-ring[**K***^α^*]The electrostatic stiffness matrix due to traveling electrostatic force[**K***^β^*]The electrostatic stiffness matrix due to traveling electrostatic force*k*The circumferential wave number of circular-ring{**Q**}The generalized force vector*r*The radius of circular-ring*T*A time-scale, 
$T=\sqrt{\rho Aa^{4}/EI}$*T̅*The period of the periodically time-varying stiffness*t*The dimensionless time, *t* = *t̂*/*T**t̂*Time*u(θ*,*t*)The dimensionless deflection of circular-ring, *u* = *û*/*g**û*( *θ,t̂*)The deflection of circular-ring, which is a function of position and time*V*The dimensionless electrical potential difference between circular-ring and traveling arc electrode, *V*^2^ = *r*^4^*ɛbV̂*^2^/*EI*g^2^*V̂*The electrical potential difference between circular-ring and traveling arc electrode{**X**}The generalized coordinates vector*α*The generalized coordinate*β*The generalized coordinate*δ*(*θ*)The Dirac delta function of *θ**ε*The permittivity of the medium in between circular-ring and traveling arc electrode*θ*The position of circular-ring in polar coordinate*ρ*The density of circular-ring*ϕ*The span angle of traveling arc electrodeΩThe dimensionless angular speed of traveling arc electrode, Ω = Ω̂*T*Ω̂The angular speed of traveling arc electrodeΩ*_cr_*The intrinsic critical speed of a traveling constant force acting on circular ring

### 2.2. Equations of Motion

As shown in Figure 1, a uniform electrical field *E̅* exists along the radial direction when there is an electrical potential difference *V̂* between the circular-ring and the traveling electrode. The uniform electrical field results in a uniform distributed electrostatic force along the radial direction between the circular-ring and the traveling electrode. The uniform distributed electrostatic force traveling around the circular-ring is expressed in terms of unit step functions

(1) $$\frac{1}{\phi }\left[H\left(\theta -(\hat{\Omega }\hat{t}-\phi /2)\right)-H\left(\theta -(\hat{\Omega }\hat{t}+\phi /2)\right)\right]\frac{ɛb{\hat{V}}^{2}}{2{\left(g-û\right)}^{2}}$$

with negligible circumferential inertia [13], the equation of motion of the circular-ring subjected to the traveling distributed force, Equation (1), is

(2) $$\rho A\frac{\partial^{2}û}{\partial {\hat{t}}^{2}}+\frac{EI}{r^{4}}\left(\frac{\partial^{4}û}{\partial \theta^{4}}+2\frac{\partial^{2}û}{\partial \theta^{2}}+û\right)=\frac{1}{\phi }\left[H\left(\theta -(\hat{\Omega }\hat{t}-\phi /2)\right)-H\left(\theta -(\hat{\Omega }\hat{t}+\phi /2)\right)\right]\frac{ɛb{\hat{V}}^{2}}{2{\left(g-û\right)}^{2}}$$

In terms of the dimensionless parameters defined in the Subsection 2.1, the equation of motion (2) is expressed in the dimensionless form

(3) $$\frac{\partial^{2}u}{\partial t^{2}}+\frac{\partial^{4}u}{\partial \theta^{4}}+2\frac{\partial^{2}u}{\partial \theta^{2}}+u=\frac{1}{\phi }\left[H\left(\theta -(\Omega t-\phi /2)\right)-H\left(\theta -(\Omega t+\phi /2)\right)\right]\frac{V^{2}}{{\left(1-u\right)}^{2}}$$

According to the basic assumption of small deflection mentioned in the beginning of this section, expand the electrostatic force term in Equation (3) by Taylor series with respect to the initial equilibrium position (*u* = 0), and neglect the second and higher-order terms, that is

(4) $$\frac{V^{2}}{{\left(1-u\right)}^{2}}\approx 1+2u$$

As a result, the equation of motion (3) can be linearized as

(5) $$\begin{array}{l} \frac{\partial^{2}u}{\partial t^{2}}+\frac{\partial^{4}u}{\partial \theta^{4}}+2\frac{\partial^{2}u}{\partial \theta^{2}}+\left\{1-\frac{2V^{2}}{\varphi }\left[H\left(\theta -\Omega t+\varphi /2\right)-H\left(\theta -\Omega t-\varphi /2\right)\right]\right\}u\\ =\frac{V^{2}}{\varphi }\left[H\left(\theta -\Omega t+\varphi /2\right)-H\left(\theta -\Omega t-\varphi /2\right)\right] \end{array}$$

The linearized equation of motion (5) is a linear partial differential equation with a time-varying coefficient. Apparently, the traveling electrical field not only provides external electrostatic force but also alters the dynamical characteristic of the micro circular-ring.

### 2.3. Discrete Equation of Motion

For the geometrical periodicity of the circular-ring, one can make an inspired guess of the deflection function,

(6) $$u(\theta ,t)=\overset{n}{\underset{k=2}{\text{∑}}}\;\left[\alpha_{k}(t)\cos k\theta +\beta_{k}(t)\sin k\theta \right]$$

where *k* is the circumferential wave number, *α_k_*(*t*) and *β_k_*(*t*) are the generalized coordinates to be determine functions of time. The term of *k* = 1 is omitted in Equation (6) because it is a rigid-body mode [11,13]. In a mathematical sense, the guessed deflection functions, cos*kθ* and sin*kθ*, represent orthogonal vectors that satisfy the boundary conditions of the circular-ring. In the cases of finite-degree-of-freedom systems, the vector space is of finite dimension and the number of vectors or natural modes is equal to the number of degrees of freedom. For continuous systems, such as a circular-ring, the number of degrees of freedom is infinite. This means that the general solution will be an infinite series. Substituting Equation (6) into Equation (5) gives

(7) $$\begin{array}{l} \overset{n}{\underset{k=2}{\text{∑}}}\;\left[{\ddot{\alpha }}_{k}(t)+\left({\left(k^{2}-1\right)}^{2}-\frac{2V^{2}}{\varphi }\left[H(\theta -\Omega t+\varphi /2)-H(\theta -\Omega t-\varphi /2)\right]\right)\alpha_{k}(t)\right]\cos k\theta \\ +\overset{n}{\underset{k=2}{\text{∑}}}\;\left[{\ddot{\beta }}_{k}(t)+\left({\left(k^{2}-1\right)}^{2}-\frac{2V^{2}}{\varphi }\left[H(\theta -\Omega t+\varphi /2)-H(\theta -\Omega t-\varphi /2)\right]\right)\beta_{k}(t)\right]\sin k\theta \\ =\frac{V^{2}}{\varphi }\left[H(\theta -\Omega t+\varphi /2)-H(\theta -\Omega t-\varphi /2)\right] \end{array}$$

Since cos*kθ* and sin*kθ* are orthogonal, like Fourier analysis, we multiply Equation (7) by cos*mθ* and sin*mθ* (*m* = 2, 3, 4, …, *n*) respectively, and integrate them over the circular-ring's circumference gives

(8) $$\begin{array}{l} \overset{n}{\underset{k=2}{\text{∑}}}\;\left[{\ddot{\alpha }}_{k}(t)+{\left(k^{2}-1\right)}^{2}\alpha_{k}(t)\right]\int_{0}^{2\pi }\cos k\theta \cos m\theta d\theta +\overset{n}{\underset{k=2}{\text{∑}}}\;\left[{\ddot{\beta }}_{k}(t)+{\left(k^{2}-1\right)}^{2}\beta_{k}(t)\right]\int_{0}^{2\pi }\sin k\theta \cos m\theta d\theta \\ -\overset{n}{\underset{k=2}{\text{∑}}}\frac{2V^{2}\alpha_{k}(t)}{\varphi }\int_{0}^{2\pi }\left[H(\theta -\Omega t+\varphi /2)-H(\theta -\Omega t-\varphi /2)\right]\cos k\theta \cos m\theta d\theta \\ -\overset{n}{\underset{k=2}{\text{∑}}}\frac{2V^{2}\beta_{k}(t)}{\varphi }\int_{0}^{2\pi }\left[H(\theta -\Omega t+\varphi /2)-H(\theta -\Omega t-\varphi /2)\right]\sin k\theta \cos m\theta d\theta \\ =\frac{V^{2}}{\varphi }\int_{0}^{2\pi }\left[H(\theta -\Omega t+\varphi /2)-H(\theta -\Omega t-\varphi /2)\right]\cos m\theta d\theta \end{array}$$

(9) $$\begin{array}{l} \overset{n}{\underset{k=2}{\text{∑}}}\;\left[{\ddot{\alpha }}_{k}(t)+{\left(k^{2}-1\right)}^{2}\alpha_{k}(t)\right]\int_{0}^{2\pi }\cos k\theta \sin m\theta d\theta +\overset{n}{\underset{k=2}{\text{∑}}}\;\left[{\ddot{\beta }}_{k}(t)+{\left(k^{2}-1\right)}^{2}\beta_{k}(t)\right]\int_{0}^{2\pi }\sin k\theta \sin m\theta d\theta \\ -\overset{n}{\underset{k=2}{\text{∑}}}\frac{2V^{2}\alpha_{k}(t)}{\varphi }\int_{0}^{2\pi }\left[H(\theta -\Omega t+\varphi /2)-H(\theta -\Omega t-\varphi /2)\right]\cos k\theta \sin m\theta d\theta \\ -\overset{n}{\underset{k=2}{\text{∑}}}\frac{2V^{2}\beta_{k}(t)}{\varphi }\int_{0}^{2\pi }\left[H(\theta -\Omega t+\varphi /2)-H(\theta -\Omega t-\varphi /2)\right]\sin k\theta \sin m\theta d\theta \\ =\frac{V^{2}}{\varphi }\int_{0}^{2\pi }\left[H(\theta -\Omega t+\varphi /2)-H(\theta -\Omega t-\varphi /2)\right]\sin m\theta d\theta \end{array}$$

By the orthogonalities of cos*mθ* and sin*mθ* [13], Equations (8) and (9) can be simplified in the matrix form,

(10) $${\left\{\text{Ẍ}\right\}}_{2(n-1)\times 1}+{\left[\text{K}\right]}_{2(n-1)\times 2(n-1)}{\left\{\text{X}\right\}}_{2(n-1)\times 1}={\left\{\text{Q}\right\}}_{2(n-1)\times 1}$$

where {**X**}, [**K**], and {**Q**} are generalized coordinates vector, stiffness matrix, and generalized force vector, respectively. The expressions of {**X**}, [**K**], and {**Q**} are

(11) $${\left\{\text{X}\right\}}^{T}=\{\begin{array}{cccccccccc} \alpha_{2}(t) & \alpha_{3}(t) & \alpha_{4}(t) & \cdots & \alpha_{n}(t) & |\beta_{2}(t) & \beta_{3}(t) & \beta_{4}(t) & \cdots & \beta_{n}(t) \end{array}\}$$

(12) $$\begin{array}{c} \begin{array}{cc} \alpha ‐\text{partition} & \;c‐\text{partition} \end{array}\\ {}[\text{K}]=\left[\begin{array}{rr} {\left[{\text{K}}^{s}\right]}_{\left(n-1)\times (n-1\right)}+{\left[{\text{K}}^{\alpha }\right]}_{\left(n-1)\times (n-1\right)} & {\left[{\text{K}}^{c}\right]}_{\left(n-1)\times (n-1\right)}\\ {\left[{\text{K}}^{c}\right]}_{\left(n-1)\times (n-1\right)}^{T} & {\left[{\text{K}}^{s}\right]}_{\left(n-1)\times (n-1\right)}+{\left[{\text{K}}^{\beta }\right]}_{\left(n-1)\times (n-1\right)} \end{array}\right]\\ \begin{array}{cc} c^{T}‐\text{partition} & \;\beta ‐\text{partition} \end{array} \end{array}$$

(13) $$\{\text{Q}\}=\left\{\begin{array}{c} {\left\{{\text{Q}}^{\alpha }\right\}}_{(n-1)\times 1}\\ {\left\{{\text{Q}}^{\beta }\right\}}_{(n-1)\times 1} \end{array}\right\}$$

The symmetrical stiffness matrix [**K**] is partitioned into four partitions named as *α*-partition, *β*-partition, *c*-partition, and the *c^T^*-partition that is the transverse of *c*-partition. The four partitions are composed of four sub-matrices [**K***^s^*], [**K***^α^*], [**K***^β^*], and [**K***^c^*]. The diagonal matrix [**K***^s^*] is the structural stiffness-matrix due to the elasticity of the circular-ring. The symmetrical matrices [**K***^α^*] and [**K***^β^*] are the electrostatic stiffness-matrices due to the traveling electrical field. The *α*-partition is the sum of [**K***^s^*] and [**K***^α^*] while the *β*-partition is the sum of [**K***^s^*] and [**K***^β^*]. The *c*-partition contains only [**K***^c^*] while the *c^T^*-partition is the transverse of [**K***^c^*]. The matrix [**K***^c^*] is also the electrostatic stiffness-matrix due to the traveling electrical field, which couples the generalized coordinates *α_k_*(*t*) and *β_k_*(*t*). Apparently, the traveling electrical field not only applies electrostatic force on the circular ring but also alter its dynamical characteristics via the electrostatic stiffness-matrices. The generalized coordinates *α_k_*(*t*) and *β_k_*(*t*) are inherently uncoupling if there is no electrical field, and could be solved independently. However, the traveling electrical field, due to [**K***^c^*], makes the generalized coordinates *α_k_*(*t*) and *β_k_*(*t*) coupling and therefore not only alter the dynamical characteristic of the structure but also further complicate the mathematical solving problem. The expressions of the elements of {**X**}, [**K**], and {**Q**} are shown in Equations (14)–(18) where *i*, *j* = 2, 3, 4, …, *n*.

(14) $$\{\begin{array}{c} K_{ii}^{s}={\left(i^{2}-1\right)}^{2},\\ K_{ij}^{s}=0,\;(i\neq j). \end{array}$$

(15) $$\{\begin{array}{l} K_{ii}^{\alpha }=-\frac{V^{2}}{\pi }-\frac{V^{2}}{\pi }\frac{\sin i\phi }{i\phi }\cos i2\Omega t,\\ K_{ij}^{\alpha }=-\frac{V^{2}}{\pi }\frac{\sin(i-j)\phi /2}{(i-j)\phi /2}\cos(i-j)\Omega t-\frac{V^{2}}{\pi }\frac{\sin(i+j)\phi /2}{(i+j)\phi /2}\cos(i+j)\Omega t,\;(i\neq j). \end{array}$$

(16) $$\{\begin{array}{l} K_{ii}^{\beta }=-\frac{V^{2}}{\pi }-\frac{V^{2}}{\pi }\frac{\sin i\phi }{i\phi }\cos(i2\Omega t+\pi ),\\ K_{ij}^{\beta }=-\frac{V^{2}}{\pi }\frac{\sin(i-j)\phi /2}{(i-j)\phi /2}\cos(i-j)\Omega t-\frac{V^{2}}{\pi }\frac{\sin(i+j)\phi /2}{(i+j)\phi /2}\cos[(i+j)\Omega t+\pi ],\;(i\neq j). \end{array}$$

(17) $$\{\begin{array}{l} K_{ii}^{c}=-\frac{V^{2}}{\pi }\frac{\sin i\phi }{i\phi }\cos(i2\Omega t+3\pi /2),\\ K_{ij}^{c}=-\frac{V^{2}}{\pi }\frac{\sin(i-j)\phi /2}{(i-j)\phi /2}\cos[(i-j)\Omega t+\pi /2]-\frac{V^{2}}{\pi }\frac{\sin(i+j)\phi /2}{(i+j)\phi /2}\cos[(i+j)\Omega t+3\pi /2],\;(i\neq j). \end{array}$$

(18) $$\{\begin{array}{l} Q_{i}^{\alpha }=\frac{V^{2}}{\pi }\frac{\sin i\phi /2}{i\phi /2}\cos i\Omega t,\\ Q_{i}^{\beta }=\frac{V^{2}}{\pi }\frac{\sin i\phi /2}{i\phi /2}\cos(i\Omega t+3\pi /2). \end{array}$$

Take a look at the elements of the three electrostatic stiffness-matrices, Equations (15)–(17), the traveling electrical field results in periodically time-varying negative stiffness and thus soften the circular-ring; this means that the issue of stabilities must be considered in design. The diagonal elements of [**K***^α^*] and [**K***^β^*] oscillate with respect to a value of −*V*^2^/*π* in the same period π/*i*Ω and same fluctuation range |*V*^2^ sin(*i*ϕ)/*i*ϕ*π*| but with a phase-angle difference of *π*. The non-diagonal elements of [**K***^α^*] and [**K***^β^*] are composed of two similar periodically time-varying functions; the first ones oscillate in phase with respect to 0 in the period 2π/|*i* − *j*|Ω and the fluctuation range $\left|\frac{V^{2}}{\pi }\frac{\sin(i-j)\phi /2}{(i-j)\phi /2}\right|$, the second ones oscillate out of phase (the phase-angle difference *π*) with respect to 0 in the period 2*π*/|*i* + *j*|Ω and the fluctuation range $\left|\frac{V^{2}}{\pi }\frac{\sin(i+j)\phi /2}{(i+j)\phi /2}\right|$. The diagonal elements of [**K***^c^*] oscillate with respect to 0 in the same period and amplitude as those of [**K***^α^*] and [**K***^β^*] but a phase-angle difference of 3*π*/2 with respect to those of [**K***^α^*]. The non-diagonal elements of [**K***^c^*] are composed of two periodically time-varying functions; the first one oscillates with respect to 0 in the same period and same amplitude as those of [**K***^α^*] and [**K***^β^*] but a phase-angle difference of *π*/2 with respect to those of [**K***^α^*]; the second one oscillates with respect to 0 in the same period and the same amplitude as those of [**K***^α^*] and [**K***^β^*] but a phase-angle difference of 3*π*/2 with respect to those of [**K***^α^*]. In summary, the fluctuation-periods (or frequencies) of the periodically time-varying electrostatic stiffness are dependent on the traveling speed Ω of the electrode, while the fluctuation ranges are not only proportional to the square of driving voltage *V*^2^ but also dependent on the span angle *ϕ* of the arc electrode; the dynamics and stabilities of the circular-ring can thus be tuned by the traveling speed Ω, span angle *ϕ*, and driving voltage *V* of the traveling arc electrode.

Equation (18) shows that the generalized forces $Q_{i}^{\alpha }$ and $Q_{i}^{\beta }$ oscillate with respect to 0 in the same period 2*π*/*i*Ω and same fluctuation-range $\left|\frac{V^{2}\sin(i\phi /2)}{\pi i\phi /2}\right|$ but a phase-angle difference of 3*π*/2. In other words, the generalized forces, Equation (18), are harmonic forces whose frequencies are dependent on the traveling speed Ω of the electrode; this means that the circular-ring will resonate if Ω approaches some critical values, which are named as critical speed Ω_cr_. However, the amplitudes (fluctuation-ranges) $\left|\frac{V^{2}\sin(i\phi /2)}{\pi i\phi /2}\right|$ of the generalized forces are not only proportional to the square of the driving voltage *V*^2^ but also dependent on the span angle *ϕ* of the arc electrode.

### 2.4. Forced Response

Equation (10) is a second-order linear ordinary differential equation system with periodical time-varying coefficients, and therefore the forced response of the circular-ring must be obtained by numerical integration. The order of Equation (10) must be reduced in advance by being transformed into state space. Here, we define the state vector as

(19) $$\begin{array}{cc} {\left\{\text{Y}\right\}}_{4(n-1)\times 1}=\left\{\begin{array}{c} \left\{\text{X}\right\}\\ \left\{\dot{\text{X}}\right\} \end{array}\right\}, & {\left\{\text{F}\right\}}_{4(n-1)\times 1}=\left\{\begin{array}{c} {\left\{\text{0}\right\}}_{2(n-1)\times 1}\\ \left\{\text{Q}\right\} \end{array}\right\} \end{array}$$

(20) $$\begin{array}{cc} {\left[\text{A}\right]}_{4(n-1)\times 4(n-1)}=\left[\begin{array}{ll} {\left[\text{0}\right]}_{2(n-1)\times 2(n-1)} & {\left[\text{I}\right]}_{2(n-1)\times 2(n-1)}\\ -[\text{K}] & {\left[\text{0}\right]}_{2(n-1)\times 2(n-1)} \end{array}\right], & {\left[\text{B}\right]}_{4(n-1)\times 4(n-1)}=\left[\begin{array}{ll} {\left[\text{0}\right]}_{2(n-1)\times 2(n-1)} & {\left[\text{0}\right]}_{2(n-1)\times 2(n-1)}\\ {\left[\text{0}\right]}_{2(n-1)\times 2(n-1)} & {\left[\text{I}\right]}_{2(n-1)\times 2(n-1)} \end{array}\right] \end{array}$$

where [**I**] is unit matrix and [**0**] is null matrix. Eventually, Equation (10) is transformed into a first-order state equation,

(21) {Ẏ}=[A]{Y}+[B]{F}

For a given initial condition {**Y**(0)}, the forced response of the circular-ring with respect to the distributed traveling electrostatic force is

(22) $$\{\text{Y}(t)\}=e^{\left[\text{A}(t)\right]}\{\text{Y}(0)\}+\int_{0}^{t}e^{\left[\text{A}(t-\tau )\right]}[\text{B}]\{\text{F}(\tau )\}d\tau$$

### 2.5. Stability Analysis

For a periodically time-varying system, the stability must be considered. Let us consider the homogeneous part of Equation (21), namely

(23) {Ẏ}=[A]{Y}

where [**A**] is a periodically time-varying matrix with the period of *T̅*. For the given 4(*n*−1) linear independent initial conditions,

(24) $${\left\{\text{Y}(0)\right\}}_{1}=\left\{\begin{array}{c} 1\\ 0\\ 0\\ \vdots \\ 0 \end{array}\right\},\;{\left\{\text{Y}(0)\right\}}_{2}=\left\{\begin{array}{c} 0\\ 1\\ 0\\ \vdots \\ 0 \end{array}\right\},\;\cdots ,\;{\left\{\text{Y}(0)\right\}}_{4(n-1)}=\left\{\begin{array}{c} 0\\ 0\\ 0\\ \vdots \\ 1 \end{array}\right\}$$

one can obtain 4(*n*-1)'s linear independent homogeneous solutions of Equation (23) in one period *T̅* by numerical integration, namely {**Y**(*T̅*) }_1_, {**Y**(*T̅*) }_2_, …, {**Y**(*T̅*) }_4(n−1)_. These linear independent homogeneous solutions compose the monodromy matrix [**C**] which is also known as state transition matrix, *i.e.*,

(25) $${\left[\text{C}\right]}_{4(n-1)\times 4(n-1)}=\left[\begin{array}{cccc} {\left\{\text{Y}(\bar{T})\right\}}_{1} & {\left\{\text{Y}(\bar{T})\right\}}_{2} & \cdots & {\left\{\text{Y}(\bar{T})\right\}}_{4(n-1)} \end{array}\right]$$

The stability is determined by the nature of the eigenvalues *λ*'s of the monodromy matrix [14,15]. The system is stable if all the eigenvalues have the magnitudes less than unity, *i.e.*, |λ|<1, unstable if at least one eigenvalue greater than unity, *i.e.*, |λ|>1, and marginally stable if at least one eigenvalue with unit magnitude and multiplicity less than unity.

## 3. Numerical Demonstration and Discussion

We adopt the first three flexural modes (*k* = 2, 3, 4) in the following numerical demonstrations because the lower-frequency modes dominate the dynamical characteristic in practice, *i.e.*,

(26) $$u(\theta ,t)=\overset{4}{\underset{k=2}{\text{∑}}}\;\left[\alpha_{k}(t)\cos k\theta +\beta_{k}(t)\sin k\theta \right]$$

The first mode (*k* = 1) is omitted because it is rigid-body mode [11,13]. The numerical integration is conducted by the commercial software MATLAB. In a mathematical sense, the natural modes represent orthogonal vectors that satisfy the boundary conditions of the ring. In cases of finite-degree-of-freedom systems, the vector space is of finite dimension and the number of vectors or natural modes is equal to the number of degrees of freedom. For continuous systems, such as ring, the number of degrees of freedom is infinite. However, in practice, the lower-frequency modes dominate the characteristics of micro devices. Therefore, three terms of modal expansion is enough for most cases.

### 3.1. Modal Analysis of the Circular-Ring

Consider the case of no electrical field, namely when the driving voltage is zero (*V* = 0) and thus [**K***^α^*] = [**K***^β^*] = [**K***^c^*] = [**0**], then Equation (10) is simplified to

(27) $$\left\{\begin{array}{c} {\ddot{\alpha }}_{k}(t)\\ {\ddot{\beta }}_{k}(t) \end{array}\right\}+\left[\begin{array}{cc} {\left(k^{2}-1\right)}^{2} & 0\\ 0 & {\left(k^{2}-1\right)}^{2} \end{array}\right]\left\{\begin{array}{c} \alpha_{k}(t)\\ \beta_{k}(t) \end{array}\right\}=\left\{\begin{array}{c} 0\\ 0 \end{array}\right\}$$

where *k* = 2, 3, 4. Assume

(28) $$\left\{\begin{array}{c} \alpha_{k}(t)\\ \beta_{k}(t) \end{array}\right\}=\left\{\begin{array}{c} {\bar{\alpha }}_{k}\\ {\bar{\beta }}_{k} \end{array}\right\}e^{i\omega_{k}t}$$

where *α̅_k_* and *β̅_k_* are the amplitudes of *α_k_*(*t*) and *β_k_*(*t*) respectively. Substituting Equation (28) into Equation (27) gives

(29) $$\left[\begin{array}{cc} {\left(k^{2}-1\right)}^{2} & 0\\ 0 & {\left(k^{2}-1\right)}^{2} \end{array}\right]\;\left\{\begin{array}{c} {\bar{\alpha }}_{k}\\ {\bar{\beta }}_{k} \end{array}\right\}=\omega_{k}^{2}\left\{\begin{array}{c} {\bar{\alpha }}_{k}\\ {\bar{\beta }}_{k} \end{array}\right\}$$

**Figure 2.**
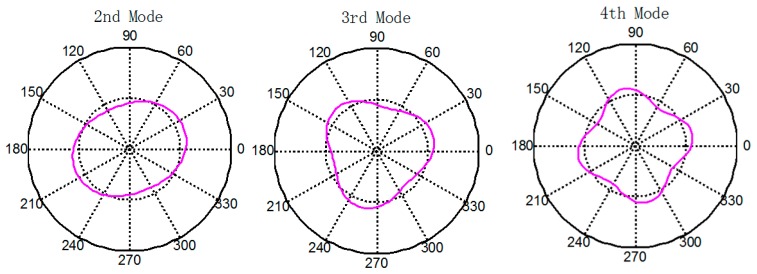
The first three flexural modes of the circular-ring.

The eigenvalues of Equation (29) are the square of the natural frequencies of the circular-ring, namely $\omega_{k}^{2}={\left(k^{2}-1\right)}^{2}$, and the corresponding eigenvectors (cos*kθ* + sin*kθ*) are the natural modes of the circular-ring. Figure 2 shows the first three flexural modes of the circular-ring.

### 3.2. The Critical Speeds of Traveling Constant Force

Consider another case of a circular-ring driven by a constant concentrated-force *F_c_* traveling around its circumference [14]. The equation of motion is

(30) $$\frac{\partial^{2}u}{\partial t^{2}}+\frac{\partial^{4}u}{\partial \theta^{4}}+2\frac{\partial^{2}u}{\partial \theta^{2}}+u=\delta (\theta -\Omega t)F_{c}$$

and Equation (10) is simplified to

(31) $$\left\{\begin{array}{c} {\ddot{\alpha }}_{k}(t)\\ {\ddot{\beta }}_{k}(t) \end{array}\right\}+\left[\begin{array}{cc} {\left(k^{2}-1\right)}^{2} & 0\\ 0 & {\left(k^{2}-1\right)}^{2} \end{array}\right]\;\left\{\begin{array}{c} \alpha_{k}(t)\\ \beta_{k}(t) \end{array}\right\}=\frac{F_{c}}{\pi }\left\{\begin{array}{c} \cos k\Omega t\\ \sin k\Omega t \end{array}\right\}$$

The particular solution of Equation (31) is

(32) $$\begin{array}{cc} \alpha_{k}(t)=\frac{F_{c}}{\pi }\frac{\cos k\Omega t}{{\left(k^{2}-1\right)}^{2}-{\left(k\Omega \right)}^{2}}, & \beta_{k}(t)=\frac{F_{c}}{\pi }\frac{\sin k\Omega t}{{\left(k^{2}-1\right)}^{2}-{\left(k\Omega \right)}^{2}} \end{array}$$

By equating the denominators in Equation (32) to zero, one can obtain the critical speeds corresponding to each mode,

(33) $${\left(k^{2}-1\right)}^{2}-{\left(k\Omega_{cr}\right)}^{2}=0\Rightarrow \Omega_{cr}^{2}=\frac{{\left(k^{2}-1\right)}^{2}}{k^{2}}=\frac{\omega_{k}^{2}}{k^{2}}$$

where *ω_k_* is natural frequencies of the circular-ring. For a circular-ring driven by a constant force traveling around its circumference, it will resonates when the traveling speed Ω approaches to the one-*k*^th^ of the natural frequency of the *k*^th^ mode. There are two square roots in (33), namely $\Omega_{cr}=\pm \left|\omega_{k}^{2}/k\right|$, one for the forward traveling wave and another the backward traveling wave; both are the same critical speed (or frequency) but have opposite wave propagation directions. The authors name the Ω_cr_s as the intrinsic critical speeds for traveling forces.

### 3.3. The Electrostatic Stiffness

By the use of three-term expansion, Equation (26), the equation of motion (10), becomes

(34) $$\begin{array}{c} \begin{array}{cc} \alpha ‐\text{partition} & \;c‐\text{partition} \end{array}\\ \left\{\begin{array}{c} \begin{array}{c} {\ddot{\alpha }}_{2}\\ {\ddot{\alpha }}_{3}\\ {\ddot{\alpha }}_{4} \end{array}\\ \begin{array}{c} {\ddot{\beta }}_{2}\\ {\ddot{\beta }}_{3}\\ {\ddot{\beta }}_{4} \end{array} \end{array}\right\}+\left[\begin{array}{cc} \begin{array}{rrr} K_{22}^{s}+K_{22}^{\alpha } & K_{23}^{\alpha } & K_{24}^{\alpha }\\ K_{23}^{\alpha } & K_{33}^{s}+K_{33}^{\alpha } & K_{34}^{\alpha }\\ K_{24}^{\alpha } & K_{34}^{\alpha } & K_{44}^{s}+K_{44}^{\alpha } \end{array} & \begin{array}{lll} \;K_{22}^{c} & \;K_{23}^{c} & \;K_{24}^{c}\\ \;K_{32}^{c} & \;K_{33}^{c} & \;K_{34}^{c}\\ \;K_{42}^{c} & \;K_{43}^{c} & \;K_{44}^{c} \end{array}\\ \begin{array}{ccc} \;K_{22}^{c} & \;K_{32}^{c} & \;K_{42}^{c}\\ \;K_{23}^{c} & \;K_{33}^{c} & \;K_{43}^{c}\\ \;K_{24}^{c} & \;K_{34}^{c} & \;K_{44}^{c} \end{array} & \begin{array}{rrr} K_{22}^{s}+K_{22}^{\beta } & K_{23}^{\beta } & K_{24}^{\beta }\\ K_{23}^{\beta } & K_{33}^{s}+K_{33}^{\beta } & K_{34}^{\beta }\\ K_{24}^{\beta } & K_{34}^{\beta } & K_{44}^{s}+K_{44}^{\beta } \end{array} \end{array}\right]\;\left\{\begin{array}{c} \begin{array}{c} \alpha_{2}\\ \alpha_{3}\\ \alpha_{4} \end{array}\\ \begin{array}{c} \beta_{2}\\ \beta_{3}\\ \beta_{4} \end{array} \end{array}\right\}=\left\{\begin{array}{c} \begin{array}{c} Q_{2}^{\alpha }\\ Q_{3}^{\alpha }\\ Q_{4}^{\alpha } \end{array}\\ \begin{array}{c} Q_{2}^{\beta }\\ Q_{3}^{\beta }\\ Q_{4}^{\beta } \end{array} \end{array}\right\}\\ \begin{array}{cc} c^{T}‐\text{partition} & \;\beta ‐\text{partition} \end{array} \end{array}$$

**Figure 3.**
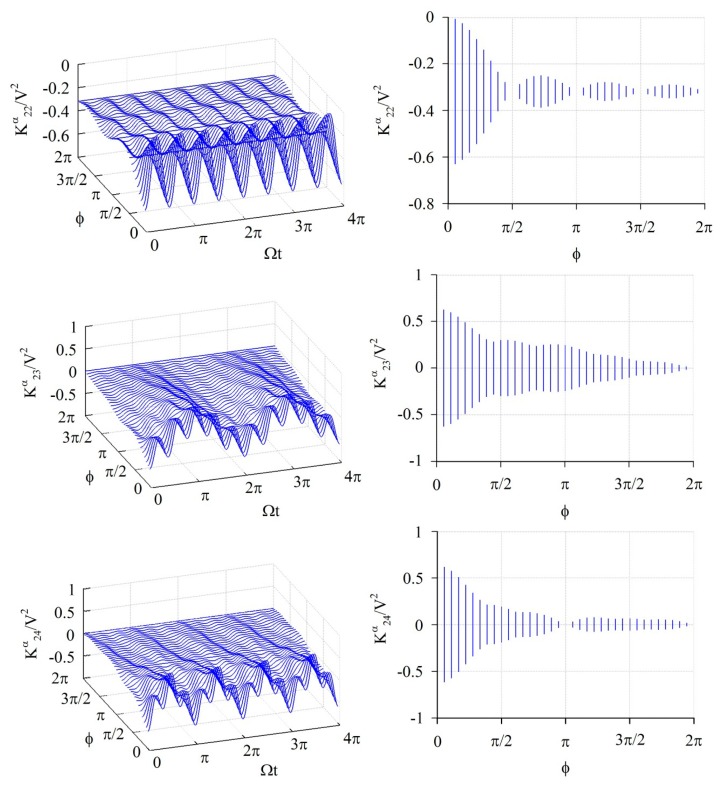
The numerical results of some feature elements ( $K_{22}^{\alpha }/V^{2}$, $K_{23}^{\alpha }/V^{2}$, and $K_{24}^{\alpha }/V^{2}$) of the *α*-partition of stiffness matrix. The graphs at the right-hand side are the projections of the graphs at the left-hand side on the plane containing *ϕ*-axis and *K*-axis.

**Figure 4.**
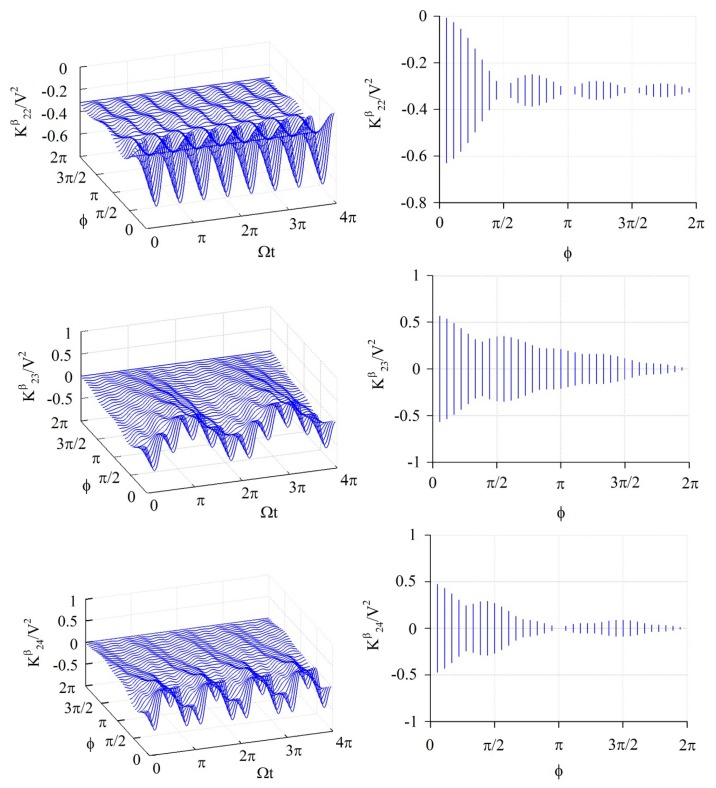
The numerical results of some feature elements ( $K_{22}^{\beta }/V^{2}$, $K_{23}^{\beta }/V^{2}$, and $K_{24}^{\beta }/V^{2}$) of the *β*-partition of stiffness matrix. The graphs at the right-hand side are the projections of the graphs at the left-hand side on the plane containing *ϕ*-axis and *K*-axis.

**Figure 5.**
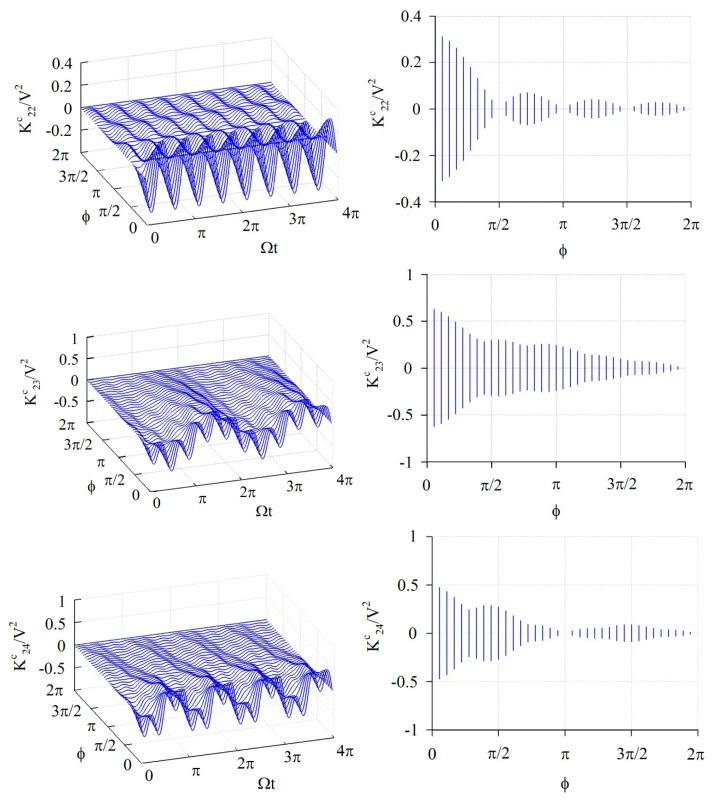
The numerical results of some feature elements ( $K_{22}^{c}/V^{2}$, $K_{23}^{c}/V^{2}$, and $K_{24}^{c}/V^{2}$) of the *c*-partition of stiffness matrix. The graphs at the right-hand side are the projections of the graphs at the left-hand side on the plane containing *ϕ*-axis and *K*-axis.

**Figure 6.**
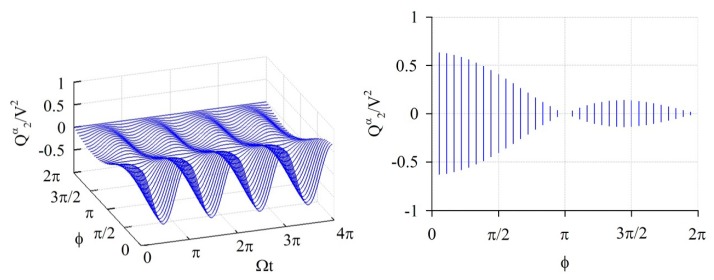
The numerical results of a feature element of generalized force vector ( $Q_{2}^{\alpha }/V^{2}$). The graph at the right-hand side are the projections of the graphs at the left-hand side on the plane containing *ϕ*-axis and *Q*-axis.

The elements of the stiffness matrix and generalized force vector are calculated by Equations (14)–(18); all of those have a periodically time-varying function. Figures 3, 4 and 5 show the numerical results of some feature elements of the stiffness matrices in the *α*-partition, *β*-partition, and *c*-partition, respectively. Figure 6 shows the numerical result of a feature element of the generalized force vector.

Figure 3 shows the numerical results of $K_{22}^{\alpha }/V^{2}$, $K_{23}^{\alpha }/V^{2}$, and $K_{24}^{\alpha }/V^{2}$. The value of $K_{22}^{\alpha }/V^{2}$ fluctuates in the period Ω*t* = *π*/2 and amplitude $\left|\frac{1}{\pi }\frac{\sin2\phi }{2\phi }\right|$, and furthermore its amplitude is 0 as *ϕ* = *κπ*/2 ≤ 2*π* where *κ* is any positive integers, namely *ϕ* = *π*/2, *π*, 3*π*/2, 2*π*. The value of $K_{23}^{\alpha }/V^{2}$ is composed of two periodic functions, one fluctuates in the period Ω*t* = 2*π*/|2 − 3| = 2*π* and amplitude $\left|\frac{1}{\pi }\frac{\sin(2-3)\phi /2}{(2-3)\phi /2}\right|=\left|\frac{1}{\pi }\frac{\sin\phi /2}{\phi /2}\right|$, and another one fluctuates in the period of Ω*t* = 2π/|2 + 3| = 2*π*/5 and amplitude $\left|\frac{1}{\pi }\frac{\sin(2+3)\phi /2}{(2+3)\phi /2}\right|=\left|\frac{1}{\pi }\frac{\sin5\phi /2}{5\phi /2}\right|$. Furthermore, the amplitude of $K_{23}^{\alpha }/V^{2}$ is 0 as *ϕ* = 2*κπ* ≤ 2*π* and *ϕ* = 2*ξπ*/5 ≤ 2*π* where *κ* and *ξ* are any positive integers, namely *ϕ* = 2*π* for the present case. The value of $K_{24}^{\alpha }/V^{2}$ is composed of two periodic functions, one fluctuates in the period of Ω*t* = 2*π*/|2 − 4| = *π* and amplitude $\left|\frac{1}{\pi }\frac{\sin(2-4)\phi /2}{(2-4)\phi /2}\right|=\left|\frac{1}{\pi }\frac{\sin\phi }{\phi }\right|$, and another one fluctuates in the period of Ω*t* = 2*π*/|2 + 4| = *π*/3 and amplitude $\left|\frac{1}{\pi }\frac{\sin(2+4)\phi /2}{(2+4)\phi /2}\right|=\left|\frac{1}{\pi }\frac{\sin3\phi }{3\phi }\right|$. Furthermore, the amplitude of $K_{24}^{\alpha }/V^{2}$ is 0 as *ϕ*; = *κπ* ≤ 2*π* and ϕ = *ξπ/*3 ≤ 2*π* where *κ* and *ξ* are any positive integers, namely *ϕ* =*π*, 2*π* for the present case.

Figure 4 shows the numerical results of $K_{22}^{\beta }/V^{2}$, $K_{23}^{\beta }/V^{2}$, and $K_{24}^{\beta }/V^{2}$. The value of $K_{22}^{\beta }/V^{2}$ fluctuates in a period of Ω*t* = *π*/2 and amplitude $\left|\frac{1}{\pi }\frac{\sin2\phi }{2\phi }\right|$, and furthermore the amplitude is 0 as *ϕ* = *κπ*/2 ≤ 2*π* where *κ* is any positive integers, namely *ϕ* = *π*/2, *π*, 3*π*/2, 2*π*. The value of $K_{23}^{\beta }/V^{2}$ is composed of two periodic functions, one fluctuates in the period of Ω*t* = 2*π*/|2 − 3| = 2*π* and amplitude $\left|\frac{1}{\pi }\frac{\sin(2-3)\phi /2}{(2-3)\phi /2}\right|=\left|\frac{1}{\pi }\frac{\sin\phi /2}{\phi /2}\right|$, and another one fluctuates in the period of Ω*t* = 2*π*/|2 + 3| = 2*π/*5 and amplitude $\left|\frac{1}{\pi }\frac{\sin(2+3)\phi /2}{(2+3)\phi /2}\right|=\left|\frac{1}{\pi }\frac{\sin5\phi /2}{5\phi /2}\right|$. Furthermore, the amplitude of $K_{23}^{\beta }/V^{2}$ is 0 as *ϕ* = 2*κπ* ≤ 2*π* and *ϕ* = 2*ξπ*/5 ≤ 2*π* where *κ* and *ξ* are positive integers, namely *ϕ* = 2*π* for the present case. The value of $K_{24}^{\alpha }/V^{2}$ is composed of two periodic functions, one fluctuates in the period of Ω*t* = 2*π*/|2 − 4| = *π* and amplitude $\left|\frac{1}{\pi }\frac{\sin(2-4)\phi /2}{(2-4)\phi /2}\right|=\left|\frac{1}{\pi }\frac{\sin\phi }{\phi }\right|$, and another one fluctuates in the period of Ω*t* = 2*π*/|2 + 4| = *π*/3 and amplitude $\left|\frac{1}{\pi }\frac{\sin(2+4)\phi /2}{(2+4)\phi /2}\right|=\left|\frac{1}{\pi }\frac{\sin3\phi }{3\phi }\right|$. Furthermore, the amplitude of $K_{24}^{\alpha }/V^{2}$ is 0 as *ϕ* = *κπ* ≤ 2*π* and ϕ = *ξπ*/3 ≤ 2*π* where *κ* and *ξ* are any positive integers, namely *ϕ* = *π*, 2*π* for the present case.

Figure 5 shows the numerical results of $K_{22}^{c}/V^{2}$, $K_{23}^{c}/V^{2}$, and $K_{24}^{c}/V^{2}$. The value of $K_{22}^{c}/V^{2}$ fluctuates in a period of Ω*t* = *π*/2 and amplitude $\left|\frac{1}{\pi }\frac{\sin2\phi }{2\phi }\right|$, and furthermore the amplitude is 0 as *ϕ* = *κπ* ≤ 2*π* where *κ* is any positive integer, namely *ϕ* = *π*/2, *π*, 3*π*/2, 2*π*. The value of $K_{23}^{c}/V^{2}$ is composed of two periodic functions, one fluctuates in the period of Ω*t* = 2*π*/|2 − 3| = 2*π* and amplitude $\left|\frac{1}{\pi }\frac{\sin(2-3)\phi /2}{(2-3)\phi /2}\right|=\left|\frac{1}{\pi }\frac{\sin\phi /2}{\phi /2}\right|$, and another one fluctuates in the period of Ω*t* = 2*π*/(2 + 3) = 2*π*/5 and amplitude $\left|\frac{1}{\pi }\frac{\sin(2+3)\phi /2}{(2+3)\phi /2}\right|=\left|\frac{1}{\pi }\frac{\sin5\phi /2}{5\phi /2}\right|$. Furthermore, the amplitude of $K_{23}^{c}/V^{2}$ is 0 as *ϕ* = 2*κπ* ≤ 2*π* and *ϕ* = 2*ξπ* ≤ 2*π* where *κ* and *ξ* are positive integers, namely *ϕ* = 2*π* for the present case. The value of $K_{24}^{c}/V^{2}$ is composed of two periodic functions, the one fluctuates in the period of Ω*t* = 2*π*/|2 − 4| = *π* and amplitude $\left|\frac{1}{\pi }\frac{\sin(2-4)\phi /2}{(2-4)\phi /2}\right|=\left|\frac{1}{\pi }\frac{\sin\phi }{\phi }\right|$, and another one fluctuates in the period of Ω*t* = 2*π*/|2 + 4| = *π*/3 and amplitude $\left|\frac{1}{\pi }\frac{\sin(2+4)\phi /2}{(2+4)\phi /2}\right|=\left|\frac{1}{\pi }\frac{\sin3\phi }{3\phi }\right|$. Furthermore, the amplitude of $K_{24}^{c}/V^{2}$ is 0 as *ϕ* = *κπ* ≤ 2*π* and *ϕ* = *ξπ*/3 ≤ 2*π* where *κ* and *ξ* are any positive integers, namely *ϕ* = *π*, 2*π* for the present case. Figure 6 shows the numerical results of $Q_{2}^{\alpha }/V^{2}$, it fluctuates in the period of Ω*t* = *π* and amplitude $\left|\frac{1}{\pi }\frac{\sin\phi }{\phi }\right|$, and furthermore the amplitude is 0 as *ϕ* = *κπ* ≤ 2*π* where *κ* is any positive integer, namely *ϕ* = *π*, 2*π* for the present case.

In summary, the traveling electrostatic force not only applies a harmonic force on the circular ring but also induces periodically time-varying electrostatic stiffness; the periodicities are dependent on the speed of the traveling electrode, while the fluctuation ranges are dependent on the driving voltage and span angle of the arc electrode, and furthermore, by tuning the span angle of the arc electrode, one can eliminate the fluctuations of some specific electrostatic stiffness.

### 3.4. Forced Response

By the use of Equations (22), (26), (34), and zero initial condition, namely {**Y**(0)} = {**0**}, one has the forced response of the circular-ring at the central location of the traveling distributed electrostatic force

(35) $$u(\Omega t,t)=\overset{4}{\underset{k=2}{\text{∑}}}\;\left[\alpha_{k}(t)\cos k\Omega t+\beta_{k}(t)\sin k\Omega t\right]$$

**Figure 7.**
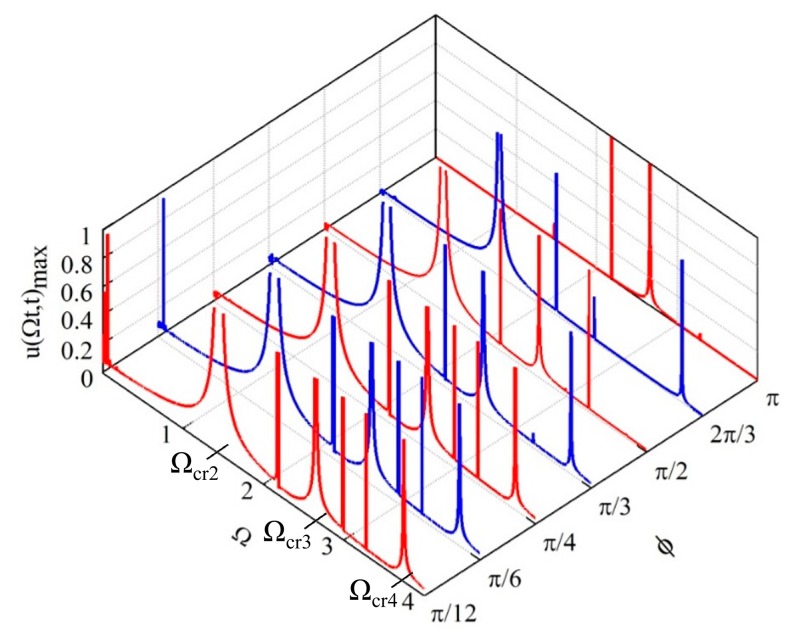
The maximum responses of the circular ring at the central point of the distributed traveling electrostatic force (*V* = 1).

Figure 7 shows the maximum response for different traveling speeds Ω and electrode span angles *ϕ*, in which there appears more peaks other than the intrinsic critical speeds Ω_cr_s. Review on the case of traveling constant force (Subsection 3.2), the forward and backward traveling waves of the circular-ring are with the same critical speed (or resonant frequency). However, for the case of traveling electrostatic force (Figure 7), there are more peaks (critical speeds) other than the intrinsic critical speeds; this phenomenon is due to the fact that the electrostatic force makes the generalized coordinates *α_k_*(*t*) and *β_k_*(*t*) coupling (view on the *c*-partition of the stiffness matrix in Equation (34)). The coupling of the generalized coordinates results in different critical speeds (or frequencies) for the forward and backward traveling waves of the circular-ring. Another interesting phenomenon is that the number of peaks is not the same for different electrode span-angle *ϕ*. This phenomenon is in agreement with the results in the Section 3.3; one can eliminate the fluctuations of some specific electrostatic stiffness by tuning the span angle of the arc electrode; especially for the case of *ϕ* = *π*, only three peaks appear at the intrinsic critical speeds (Ω_cr2_, Ω_cr3_, and Ω_cr4_,) because the electrostatic stiffness is eliminated.

### 3.5. Stability

**Figure 8.**
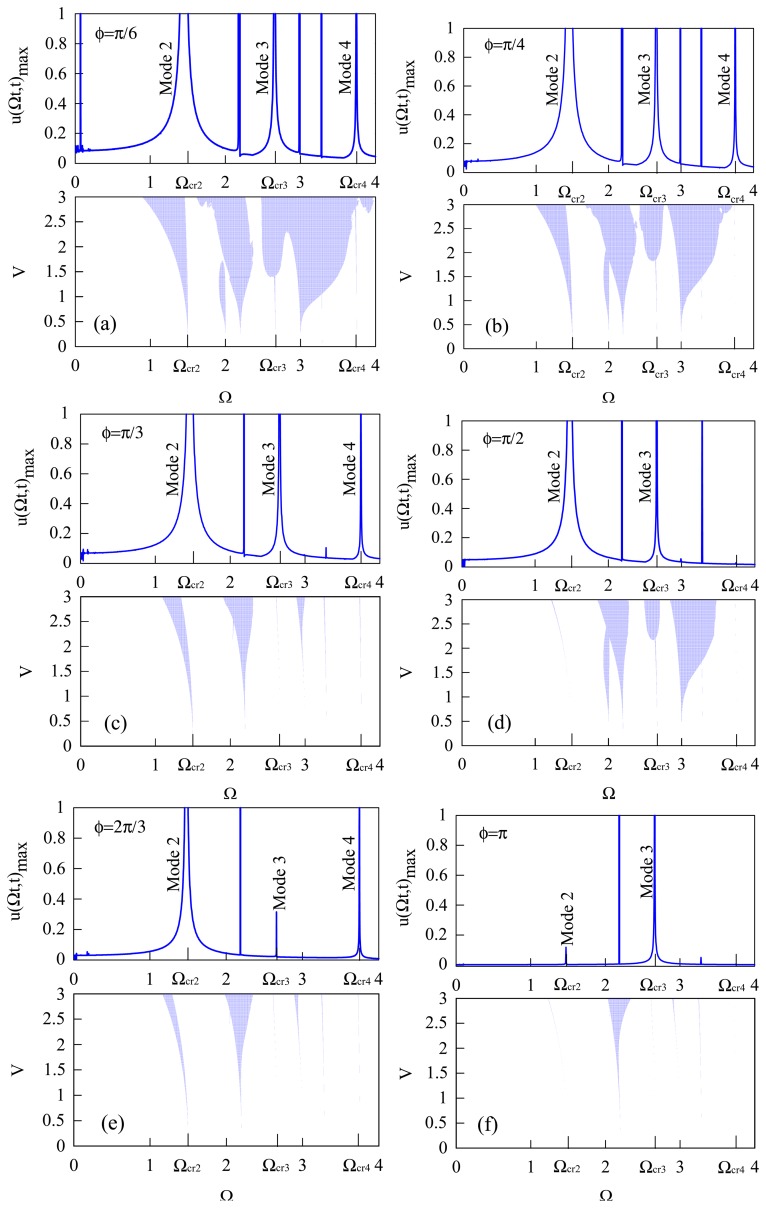
The stabilities of the circular-ring with respect to driving voltage *V* and traveling speed Ω for different electrode span angle *ϕ*. The upper parts of Figure (**a**)–(**f**) is the maximum responses of the circular ring at the central point of the distributed traveling electrostaic (*V* = 1), while the lower part of each figure shows the instable region of the circular-ring with respect to driving voltage V and traveling speed Ω.

By using the stability analysis method in Subsection 2.5 and the three-term modal expansion, Equation (26), Figure 8a–f shows the forced response (the upper one) and instable regions (the lower one) of the circular-ring driven by the traveling electrodes with different electrode span-angles *ϕ* (π/6, π/4, π/3, π/2, 2π/3, π, *etc.*). The instable regions appear near the critical speeds and furthermore expand with increasing driving voltage. The instable regions can be eliminated by tuning the electrode span angle *ϕ*.

## 4. Conclusions

An analytical model is derived for simulating the electromechanical behavior of a micro circular-ring around which goes an arc electrode. Some interesting phenomena are addressed. The traveling electrode not only applies electrostatic force on the circular-ring but also alters its dynamical characteristics via the electrostatic stiffness-matrices. The traveling electrical field results in periodically time-varying negative stiffness and thus softens the circular-ring structure. It is known that, when a structure is subjected to a traveling constant force, its natural mode will be resonated as the speed of the traveling constant force approaches a critical value—namely the critical speed—and each natural mode refers to exactly one critical speed. However, for the case of a traveling electrostatic force, the number of critical speeds is more than that of the natural modes. This is due to the fact that the traveling electrostatic force makes the resonant frequencies of the forward and backward traveling waves of the circular-ring different. Another interesting phenomenon is that the resonance and stability can be controlled by the span-angle of the traveling electrode though the electrostatic force alters the dynamics and stabilities of microstructures. This paper derives an analytical model for simulating a micro-ring driven by a traveling piecewise-electrode, which extends the fundamental insights into the electromechanical behavior of microstructures driven by electrostatic forces as well as the future development of MEMS/NEMS devices with electrostatic actuation and sensing.
